# Next-Generation Sequencing Analysis Reveals Frequent Familial Origin and Oligogenism in Congenital Hypothyroidism With Dyshormonogenesis

**DOI:** 10.3389/fendo.2021.657913

**Published:** 2021-06-24

**Authors:** Isabelle Oliver-Petit, Thomas Edouard, Virginie Jacques, Marie Bournez, Audrey Cartault, Solange Grunenwald, Frédérique Savagner

**Affiliations:** ^1^ Endocrine, Genetics, Bone Diseases, and Paediatric Gynecology Unit, Children’s Hospital, CHU Toulouse, Toulouse, France; ^2^ Inserm UMR 1048, Institute of Metabolic and Cardiovascular Diseases (I2MC), Toulouse, France; ^3^ Biochemistry and Genetic Laboratory, Federative Institute of Biology, CHU Toulouse, Toulouse, France; ^4^ Pediatric Unit, Hospital Le Bocage, CHU Dijon, Dijon, France; ^5^ Department of Endocrinology and Metabolic Diseases, Cardio-Vascular and Metabolic Unit, CHU Larrey, Toulouse, France

**Keywords:** high throughput molecular screening, familial origin, oligogenicity, congenital hypothyroidism, thyroid dyshormonogenesis

## Abstract

**Context:**

Congenital hypothyroidism (CH) is related to dyshormonogenesis in 15% to 40% of the world population and associated with homozygous or heterozygous variants in the main genes of the hormone synthesis pathway. Emerging diagnostic tools, such as next-generation sequencing (NGS), have been used to efficiently explore panels of genes and identify complex mechanisms of pathogenesis.

**Objective:**

We explored 19 candidate genes known to be causative for permanent or transient CH to evaluate the role of complex gene variations in CH phenotype.

**Patients, Design and Setting:**

Using the NGS approach, we studied 65 newborns with thyroid dyshormonogenesis (TDH). New variants were assessed *in silico* for pathogenicity.

**Results:**

Among the 65 infants, 56.9% presented a variant in one or more genes of the thyroid hormone synthesis axis. We identified homozygous or compound heterozygous variants in the *TG*, *DUOX2*, *TPO*, or *SLC5A5* genes in 10 infants and heterozygous variants in *DUOX2*, *TG*, *TPO*, and *TSHR* in 19 others. In seven cases, a heterozygous variant in the *TG* gene was the unique anomaly detected, but related to disturbed hormonal balance. Oligogenic variants were found in eight infants associated with severe CH and goiter in five of them.

**Conclusion:**

The systematic exploration of genes involved in thyroid hormone synthesis by NGS in TDH showed high diagnostic relevance. Oligogenic inheritance could be related to phenotypic heterogeneity and a high frequency of goiter.

## Introduction

Congenital hypothyroidism (CH) is the most common inborn endocrine disorder detected at birth by mass biochemical newborn screening. Epidemiology for CH has recently changed with an increased incidence close to 1/2,600 in France, mainly related to an increase in CH with thyroid dyshormonogenesis (TDH) representing 37% to 49% of CH cases relative to regional origin (1–3). In the south of France, 47% of CH are due to TDH and 53% to thyroid dysgenesis (TD). Genetic exploration of CH has often been restricted to a small number of genes because of a cost and time-consuming process to explore them by Sanger technology. Apart from a monogenic pattern of inheritance, the role of oligogenicity in disease development remains poorly explored. Recent studies have used next-generation sequencing (NGS) technology for better genotyping of newborns with suspected CH (4–9). Despite differences in the methodological approach, all studies highlighted an increased proportion of familial origin single or multiple pathogenic variants leading to thyroid dyshormonogenesis.

In France, the nationwide neonatal screening program of CH, implemented since 1978, identifies without ethnic *a priori* assumptions all children with high TSH levels. They are explored in accordance with the European Society for Paediatric Endocrinology (ESPE) consensus by biology, ultrasonography, and scintigraphy to distinguish aetiologies between TD and TDH. Analysis of this large and multi-ethnic unselected population could specify the landscape of familial aetiology in TDH development while considering the frequency of transient CH. The present NGS study established a targeted thyroid gene panel in a subset of 65 children screened at birth and confirmed to have CH. The aim of our study was to explore the frequency of complex molecular mechanisms for TDH related to phenotypic heterogeneity. We focused on TDH due to its increased incidence in our region (+7.9% per year) compared to the stable incidence of TD.

## Patients and Methods

### Patients

Sixty-five patients were included in the study (30 females and 35 males); all except one were issued from non-related families living in the Midi-Pyrenees’ region (south west of France, 3 million inhabitants). In 10% (7/65), different ethnic origins were present (Turkish, Maghreb countries).

All patients had early hypothyroidism as defined by ESPE consensus guidelines for congenital hypothyroidism screened at birth (regional neonatal screening), confirmed at the age of 10 days, and explored by ultrasonography (10). Secondary to genetic sampling, scintigraphy was performed to confirm aetiologies and for three infants to redirect toward TD (F37 for ectopia and F6, F14 for hypoplasia). Ectopy is a rare event in our population, as only 16% of TD presented ectopia during the same period of this study. For F2 patient, radionuclide scintigraphy revealed severely reduced uptake by the thyroid gland suggesting a functional defect of NIS. Based on serum FT4 levels, CH was defined as severe when FT4 was <5 pmol/L, moderate when FT4 was 5 to 10 pmol/L, and mild when FT4 was 10 to 15 pmol/L. TSH resistance was referred as FT4 in the normal range with a high level of TSH.

Infants were treated from diagnosis by levothyroxine (5–15 µg/kg/d) and re-evaluated after two or three years as proposed by consensus: 44 (67%, 44/65) were confirmed as persistent TDH; the 21 remaining infants had transient CH, but for seven of them, the biological profile showed persistent high levels of TSH and normal FT4. Relative to FT4 values, treatment was stopped for infants with transient CH. For 27 children (41.5%, 27/65), clinical goiter was suspected and confirmed by ultrasonography.

For all families except five (F22, F31, F33, F36, and F 37), samples were obtained from family members to check for inheritance and co-segregation with phenotype. Written informed consent for patients or relatives was obtained for NGS and/or Sanger analyses and data were included in a biocollection (number: DC-2015-2450).

### DNA Extraction and Sequencing

Genomic DNA was isolated from peripheral blood using the QIAamp DNA blood kit according to the manufacturer’s instructions (Qiagen, Hilden, Germany). A total of 10 ng of DNA per sample was used for library preparation, using the DNAprep with enrichment protocol (Illumina; San Diego, CA) and a custom NGS panel including in 19 thyroid-related genes (**Supplemental Table 1**). Sequencing was performed on the Nextseq550 platform (Illumina) using the Mid Output kit v2.5. For a sequence variant to be considered valuable, a sequencing coverage of 30× reads was used as a minimum requirement in the present study. Sequence data were processed using custom bioinformatic software and compared to NextGENe independent analysis (Softgenetics, State College, PA, USA) to align reads to the HG19 reference genome. Copy number variants (CNVs) were detected using the COV’COP tool (11) and confirmed by MLPA analysis (MRC-Holland, Amsterdam, Netherlands). All reported variants, described using the HGVS nomenclature, were explored using public and license databases, including HGMD professional, gnomAD, and ClinVar, as well as literature searches. Minor allele frequency <5% was considered. The majority of variants were rated according to the American College of Medical Genetics and Genomics (ACMG) guidelines (12) and VarSome ACMG implementation (http://varsome.com). For variants with no pathogenic reporting, we classified them based on allele frequency in corresponding ethnic population, cosegregation with thyroid balance within the upper limit (TSH value) for the carrier parent and non-published data. Amino acid predictions were performed using the MutationTaster (http://www.mutationtaster.org), SIFT (http://sift.jvci.org), and PolyPhen-2 (http://genetics.bwh.harvard.edu/pph2) software tools and additional supporting evidence for pathogenicity was stated for variants of uncertain significance (VUS). We considered variants as deleterious when concordant annotations by two of these software tools using different algorithms were present. For intronic variants close to the splicing site, we used the ESEFinder2.0 software tool. Variants detected by the Illumina platform were confirmed by targeted Sanger sequencing on an ABIXL3500 apparatus using the BigDye Terminator v3.1 Cycle Sequencing kit (Applied Biosystems; Thermo Fisher Scientific, Inc.).

The data for this study have been deposited in the European Nucleotide Archive (ENA) at EMBL-EBI under accession number PRJEB42793.

### Statistical Analysis

The statistical significance was assessed using the Mann-Whitney *U* test. Differences were considered significant at *P* < 0.05. All analyses were performed using StatView, version 5.0 (SAS Institute, Gary, NC, USA).

## Results

### Spectrum of Genetic Variants

Among the 65 children, 37 (56.9%) presented a constitutive variant from different ACMG classes: 3 (VUS), 4 (probably pathogenic), and 5 (pathogenic) in one or two genes of the thyroid hormone biosynthesis pathway, according to VarSome results and, for some variants, to our own pathogenicity evidence as referred in *Patients and Methods* section. Functional studies will ultimately be required to prove the pathogenicity of novel variants. We showed that for the majority of patients (78.4%; 29/37), variants belonging to classes 4 and 5 have been previously reported in databases and/or in the literature. Biallelic variants in *TG*, *TPO, DUOX2,* and *SLC5A5* genes were found in 27% (10/37) of patients, whereas monoallelic variants in *TSHR*, *TPO, DUOX2,* and *TG* were identified in 51.3% (19/37). Oligogenism was identified for 21.6% (8/37) of the infants, mainly involving variants in the *TG* and *TPO* or *DUOX2* genes, except for homozygotic twins (F32, *DUOX2/TPO*). Considering mono or biallelic variants in a unique gene independently of oligogenic inheritance (29/37), the *TG* gene was most commonly associated with TDH in our cohort (37.9%; 11/29), followed by a fairly equivalent percentage of variants in the *TPO* (24.1%; 7/29) and *DUOX2* (20.7%; 6/29) genes. For *TG*, six variants (F1, F4, F24, F28, F31, and F33) led to truncated TG before or in the carboxy-terminal acetyl cholinesterase-like domain, which is crucial for conformation and intracellular trafficking (13, 14).

According to ACMG class 3, VUS corresponded to monoallelic variants in the *TG* (F3, F4, F9, F13, and F29), *TPO* (F27), and *DUOX2* (F9) genes. For F3 and F4, VUS in *TG* were associated with pathogenic *TG* variants. For F9, two VUS were associated (in *TG* and *DUOX2*) and related to neonatal goiter. The latest two VUS in the *TG* gene (F13, F29) were related to moderate CH in a 27-week premature girl without goiter (F13) and to mild CH in a child presenting with psychomotor delay (F29). Finally, for F27, the splicing variant was related to homozygous deletion of exon 17 of the *TPO* gene, confirmed by MLPA analysis (data not shown).

We did not detect any variants in the *DUOX1*, *DUOXA1,* and *GNAS1* genes, according to the low frequency of these events in familial TDH (15, 16). No pathogenic variants in other genes (coding and non-coding regions) explored in our study and related to dysgenesis and dysthyroidism were identified.

Genotypical data and inheritance for the 37 children positive for ACMG classes 3 to 5 of the constitutive variants are shown in **Table 1**.

**Table 1:** Genotypical data and inheritance for the 37 children diagnosed for TDH.

ID	Genes	exon/Intron	Nucleotide	Protein (zygosity)	dbSNP number	gnomAD	Pathogeny	Maternal	Paternal	SIFT	Polyphen-2	MutationTaster	Improved classification	Causative ACGM level	HGMD- First report
						MAF		Genotype	Genotype	score	score	prediction	according to ACGM guidelines		
F11	*DUOX2*	E22	c.2895_2898del	p.Phe966Serfs*29 (Het)	rs530719719	0.002	P	WT	Het	0	1	Disease-causing	PVS1+PP3+PP5	5	Moreno, 2002, N Engl J Med
F12	*DUOX2*	E22	c.2895_2898del	p.Phe966Serfs*29 (Het)	rs530719719	0.002	P	Het	WT	0	1	Disease-causing	PVS1+PP3+PP5	5	Moreno, 2002, N Engl J Med
F21	*DUOX2*	E22	c.2895_2898del	p.Phe966Serfs*29 (Het)	rs530719719	0.002	P	Het	WT	0	1	Disease-causing	PVS1+PP3+PP5	4	Moreno, 2002, N Engl J Med
F30	*DUOX2/DUOX2*	E5	c.498C>A	p.Ser166Arg (Homo)	rs370438048	0.0001	LP	Het	Het	0	1	Disease-causing	PM1+PM2+PP3+PP4	4	N/A
F36	*DUOX2*	I26	c.3515+5 G>T	p.? (Het)	rs375943962	0.00	LP	N/A	N/A	0	1	Disease-causing	PM2+PP3+PP4+PP5	4	N/A
F37	*DUOX2*	E20	c.2635G>A	p.Glu879Lys (Het)	rs774556391	0.00	LP	N/A	N/A	0	1	Disease-causing	PM1+PM2+PP3+PP5	4	Maruo, 2008, J Clin Endocrinol Metab
F32-1	*DUOX2/TPO*	E29 (*DUOX2*)	c.3799C>T	p.Arg126Trp (Het oligo)	rs752437461	0.00	LP	Het	WT	0	1	Disease-causing	PM1+PM2+PP3+PP5	4	Wang, 2014,Clin Endocrinol
		E8 (*TPO*)	c.1199T>A	p.Val400Asp (Het oligo)	N/A	N/A	LP	WT	Het	0	1	Disease-causing	BP1+PM2+PP3+PP4+PP5	4	N/A
F32-2	*DUOX2/TPO*	E29 (*DUOX2*)	c.3799C>T	p.Arg126Trp (Het oligo)	rs752437461	0.00	LP	Het	WT	0	1	Disease-causing	PM1+PM2+PP3+PP5	4	Wang, 2014,Clin Endocrinol
		E8 (*TPO*)	c.1199T>A	p.Val400Asp (Het oligo)	N/A	N/A	LP	WT	Het	0	1	Disease-causing	BP1+PM2+PP3+PP4+PP5	4	N/A
F2	*SLC5A5/SLC5A5*	E13	c.1593C>G	p.Tyr531* (Homo)	rs121909177	0.00	P	Het	Het	0	1	Disease-causing	PVS1+PM2+PP3+PP5	5	Pohlenz, 1998, J Clin Invest
F13	*TG*	E21	c.4481C>T	p.Pro1494Leu (Het)	rs146498231	0.0008	VUS	Het	WT	0	0.75	Disease-causing	BP1+PM2+PM3	3	Makretskaya, 2018,PLoS One
F16	*TG*	I30	c.5686+1G>A	p.? (Het)	rs374620255	0.00	P	Het	WT	0	0.9	Disease-causing	PVS1+PM2+PP3	5	Hermanns, 2013, J Pediatr Endocrinol Metab
F18	*TG*	E29	c.5485C>T	p.Arg1829Trp (Het)	rs148982115	0.0002	LP	WT	Het	0	0.9	Disease-causing	PM2+PP3+PP4+PP5	4	N/A
F24	*TG*	E11	c.2787del	p.Lys929Asnfs*37 (Het)	N/A	N/A	LP	WT	Het	0	1	Disease-causing	PVS1+PM2	4	N/A
F29	*TG*	E44	c.7640T>A	p.Leu2547Gln (Het)	rs2979042	0.0001	VUS	WT	Het	0	0.95	Disease-causing	BP1+PP3+PP4+PP5	3	Nicholas, 2016, J Clin Endocrinol Metab
F31	*TG*	E22	c.4588C>T	p.Arg1530* (Het)	rs121912646	0.0001	P	N/A	N/A	0	1	Disease-causing	PVS1+PM2+PP3+PP5	5	Targovnik, 1993,J Clin Endocrinol Metab
F33	*TG*	E30	c.5676G>A	p.Trp1892* (Het)	N/A	N/A	P	N/A	N/A	0	1	Disease-causing	PVS1+PM2+PP3	5	N/A
F10	*TG/DUOX2*	E9 (*TG*)	c.1958G>A	p.Gly653Asp (Het oligo)	rs2069548	0.010	LP	WT	Het	0	0.99	Disease-causing	PM2+PP3+PP4+PP5	4	de Filippis, 2017, Hum Mol Genet
		E31 (*DUOX2*)	c.4156G>A	p.Gly1386Ser (Het oligo)	rs139584933	0.0001	LP	Het	WT	0	1	Disease-causing	PM2+PP3+PP4+PP5	4	Tan, 2016, Horm metab Res
F20	*TG/DUOX2*	E3 (*TG*)	c.199G>A	p.Gly67Ser (Het oligo)	rs116340633	0.010	P	Het	WT	0	1	Disease-causing	BP1+PP3+PP4+PP5	3	de Filippis, 2017, Hum Mol Genet
		E31 (*DUOX2*)	c.2895_2898del	p.Phe966Serfs*29 (Het oligo)	rs530719719	0.002	P	WT	Het	0	1	Disease-causing	PVS1+PP3+PP5	5	Moreno, 2002, N Engl J Med
F7	*TG/DUOX2*	E10 (*TG*)	c.2381G>T	p.Gly794Val (Het oligo)	rs1180105954	0.00	LP	Het	WT	0	1	Disease-causing	PP1+PP3+PP4+PP5	4	N/A
		E20 (*DUOX2*)	c.2597T>G	p.Met866Arg (Het oligo)	rs200948626	0.0001	LP	WT	Het	0	1	Disease-causing	PM1+PM2+PP3+PP4+PP5	4	Muzza, 2013, J Clin Endocrinol Metab
F9	*TG/DUOX2*	E27 (*TG*)	c.5299_5301del	p.Asp1767del (Het oligo)	rs112749206	0.0003	VUS	Het	WT	0	1	Disease-causing	BP1+PP3+PP4+PP5	3	N/A
		E25 (*DUOX2*)	c.3367G>A	p.Ala1123Thr (Het oligo)	rs113632824	0.0001	VUS	WT	Het	0.09	1	Disease-causing	BP1+PP3+PP4+PP5	3	Kim, 2013,Int J Ped Endocrinol
F1	*TG/TG*	E47	c.8131A>T	p.Lys2711* (Homo)	N/A	N/A	P	Het	Het	0	0.99	Disease-causing	PVS1+PM2+PP3+PP5	5	N/A
F28	*TG/TG*	E7	c.886C>T	p.Arg296* (Homo)	rs121912648	0.0004	P	Het	Het	0	1	Disease-causing	PVS1+PM2+PP3+PP5	5	Van de Graaf, 1999, J Clin Endocrinol Metab
F3	*TG/TG*	E3	c.229G>A	p.Gly77Ser (Comp Het)	rs1422698837	0.0007	VUS	Het	WT	0	1	Disease-causing	BP1+PP3+PP4+PP5	3	Van de Graaf, 1999,J Clin Endocrinol Metab
		E41	c.7123G>A	p.Gly2375Arg (Comp Het)	rs137854434	0.0002	LP	WT	Het	0	1	Disease-causing	BP1+PM2+PP3+PP4+PP5	4	Van de Graaf, 1999, J Clin Endocrinol Metab
F4	*TG/TG*	E3	c.229G>A	p.Gly77Ser (Comp Het)	rs1422698837	0.0007	VUS	WT	Het	0	1	Disease-causing	BP1+PP3+PP4+PP5	3	Van de Graaf, 1999, J Clin Endocrinol Metab
		E4	c.416G>A	p.Trp139*(Comp Het)	rs141306917	0.0001	P	Het	WT	0	1	Disease-causing	PVS1+PM2+PP3+PP5	5	Hishinuma, 2006, J Clin Endocrinol Metab
F23	*TG/TPO*	E9 (*TG*)	c.1958G>A	p.Gly653Asp (Het oligo)	rs2069548	0.015	LP	Het	WT	0	0.99	Disease-causing	PM2+PP3+PP4+PP5	4	de Filippis, 2017, Hum Mol Genet
		E13 (*TPO*)	c.2386G>A	p.Asp796Asn (Het oligo)	rs773759871	0.00	P	WT	Het	0	1	Disease-causing	PVS1+PM1+PM2+PM5+PP3	5	N/A
F8	*TG/TPO*	E10 (*TG*)	c.2610G>T	p.Glu870His (Het oligo)	rs2229843	0.0042	LP	WT	Het	0	0.96	Polymorphism	PM2+PP3+PP4+PP5	4	N/A
		E14 (*TPO*)	c.2513G>A	p.Cys838Tyr (Het oligo)	N/A	N/A	LP	Het	WT	0	0.99	Disease-causing	PM1+PM2+PP3+PP4	4	N/A
F22	*TPO*	E9	c.1399G>A	p.Val467Met (Het)	rs373267637	0.0002	LP	N/A	N/A	0.01	0.94	Polymorphism	PM2+PP3+PP4+PP5	4	N/A
F25	*TPO*	E9	c.1399G>A	p.Val467Met (Het)	rs373267637	0.0002	LP	WT	Het	0.01	0.94	Polymorphism	PM2+PP3+PP4+PP5	4	N/A
F5	*TPO*	E14	c.2422del	p.Cys808Alafs*24 (Het)	rs763662774	0.0001	P	WT	Het	0	1	Disease-causing	FVS1+PM2+PP3+PP5	5	Bakker, 2000, J Clin Endocrinol Metab
F19	*TPO/TPO*	E11	c.1978C>G	p.Gln660Glu (Comp Het)	rs121908088	0.0002	P	Het	WT	0	1	Disease-causing	BP1+PM2+PP3+PP5	5	Santos, 1999, Clin Endoc
		E14	c.2395G>C	p.Glu799Gln(Comp Het)	N/A	N/A	LP	WT	Het	0	1	Disease-causing	BP1+PM1+PM2+PM5+PP3	4	Bikker, 1995, Hum Mut
F26	*TPO/TPO*	E8	c.1197G>C	p.Ser398Thr (Homo)	N/A	N/A	P	Homo	WT	0	0.94	Disease-causing	BP1+PM2+PM3+PP3+PP5	5	N/A
F27	*TPO/TPO*	E17	c.2749-2_2802del	p.? (Homo)	N/A	N/A	VUS	Het	Het	0	1	Disease-causing	BP1+PP3+PP4+PP5	3	N/A
F35	*TPO/TPO*	E5	c.387del	p.Asn129Lysfs*80 (Homo)	rs766399662	0.00	P	Het	Het	0	1	Disease-causing	FVS1+PM2+PP3+PP5	5	Rivolta, 2003, Hum Mut
F14	*TSHR*	E2	c.202C>T	p.Pro68Ser (Het)	rs142063461	0.0004	P	Het	WT	0	1	Disease-causing	BP1+PM2+PP3+PP4+PP5	5	Tenenbaum-Rakover, 2009, J Clin Endocrinol Metab
F15	*TSHR*	E11	c.1349G>A	p.Arg450His (Het)	rs189261858	0.0003	P	Het	WT	0	1	Disease-causing	BP1+PM2+PP3+PP4+PP5	5	Tenenbaum-Rakover, 2015, Thyroid
F34	*TSHR*	E11	c.1612G>A	p.Ala538Thr (Het)	rs151264748	0.00	LP	WT	Het	0	1	Disease-causing	BP1+PM2+PP3+PP4	4	N/A
F6	*TSHR*	E11	c.1349G>A	p.Arg450His (Het)	rs189261858	0.0003	P	Het	WT	0	1	Disease-causing	BP1+PM2+PP3+PP4+PP5	5	Tenenbaum-Rakover, 2015, Thyroid

WT, wild type; homo, homozygous; Het, heterozygous; Comp Het: compound heterozygous; Het Oligo, oligogenism; P, pathogenic; LP, likely pathogenic; VUS, variant of uncertain significance; TG, thyroglobulin; TPO, thyroperoxidase; TSHR, TSH receptor; SLC5A5, sodium/iodide symporter (NIS); DUOX2, dual oxidase 2; N/A, not applicable.

### Phenotype-Genotype Correlation

**Figure 1:**
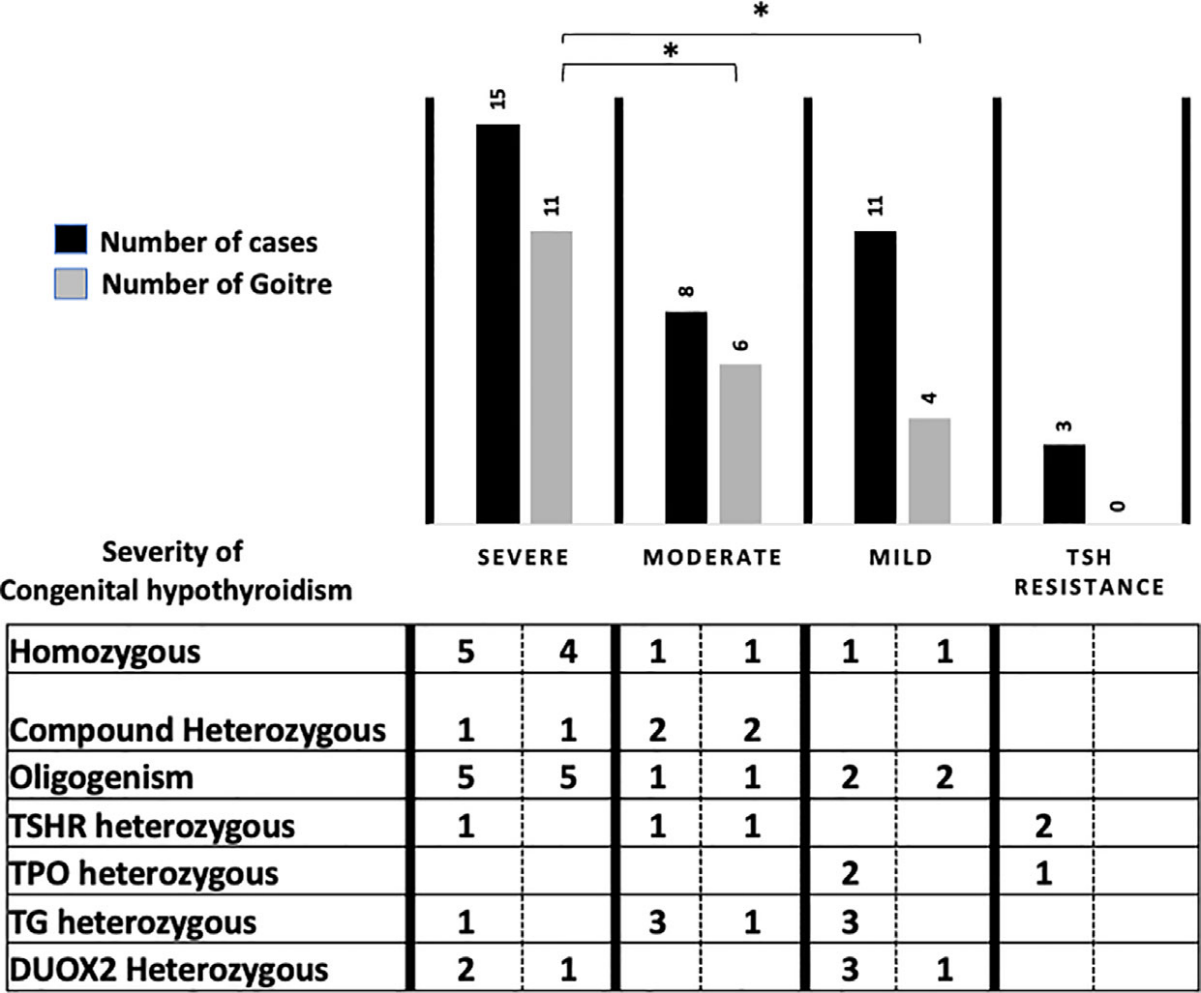
Phenotype/genotype correlation for the 37 patients according to severity of Congenital Hyperthyroidism (ESPE guidelines), NGS results and related to type and number of variants. *p < 0.05.

Among the 37 children presenting constitutive variants, 21/37 (55.5%) were male with an increased male/female sex ratio of 1.31 (21/16) compared to the global sex ratio of 1.18 (35/30) or to that of the NGS negative group of 1.15 (15/13). No extra-thyroidal phenotype has been noticed for all the children. Goiter was significantly associated with positive cases (56.7%, 21/37) compared to infants negative for variants but with proven CH, (14.2%, 4/28). Goiter was also significantly associated with homozygous, compound heterozygous and oligogenic variants regardless of the severity of CH (94% 17/18). Types of variants related to CH severity and goiter for the 37 infants are presented in **Figure 1**.

Pathogenic variants or VUS were associated with permanent TDH for 89.1% (33/37) and with transient hypothyroidism in 10.8% (4/37), all of the latter associated with *DUOX2* variants. For 75% of the *TSHR*-positive cases (F6, F14, F15, F34), no goiter was detected, but hypoplasia was noticed in two infants after scintigraphy. Two cases corresponded to the same heterozygous variant Arg450His with a difference in the biological spectrum from TSH resistance for F6 to severe hypothyroidism for F15.

Comparing patient phenotypes using the ESPE criteria for CH severity based on FT4 levels, biallelic and oligogenic variants were associated with severe CH (73.3%, 11/15) and goiter 90.9%, 10/11), while heterozygous variants in the *TG*, *TPO, DUOX2,* and *TSHR* genes were associated with infants presenting moderate and mild CH (63.2%, 12/19) with a low risk of goiter (25%, 3/12).

We also identified a new homozygous deletion in the TPO gene in one infant (F27) presenting moderate CH with goiter. Among the diverse splicing mRNA TPO variants already described, none were related to the unique absence of exon 17 (17). Even if the structure of TPO remains to be determined, a recent modelling study suggested that homozygous loss of the intracellular domain of this enzyme is responsible for cell trafficking and could impact the conformation of its active site (18).

Exploring for the role of heterozygous *TG* VUS in CH (F13 and F29), we showed that for F13 with CH in a 27-week premature girl, the mother presented with clinical adult-onset goiter and moderate hypothyroidism associated with the same heterozygous variant in the *TG* gene. For F29, the father of a boy with mild CH had a history of hypothyroidism and goiter with adult onset, related to the same VUS. Clinical and inheritance data for the 37 infants are shown in **Table 2**. Clinical and biological data for the 28 remaining infants without identified variant are listed in **Supplemental Table 2**.

**Table 2:** Clinical and biological data for the 37 children diagnosed for TDH.

ID	Sex	Serum TSH	Serum FT4	Thyroid	CH Severity	Zygosity	Gene	Permanent/transient
		(confirmation)		Evaluation				
F11	F	109	3.4	Goitre	Severe	Het	*DUOX2*	Transient
F12	F	92	12	Eutopic	Mild	Het	*DUOX2*	Transient
F21	M	74	15	Eutopic	Mild	Het	*DUOX2*	Transient
F30	M	>300	3.4	Goitre	Severe	Homo	*DUOX2/DUOX2*	Permanent
F36	F	76	13	Goitre	Mild	Het	*DUOX2*	Transient
F37	F	640	2.6	Ectopia	Severe	Het	*DUOX2*	Permanent
F32-1	M	227	3.5	Goitre	Severe	Oligo	*DUOX2/TPO*	Permanent
F32-2	M	327	3.5	Goitre	Severe	Oligo	*DUOX2/TPO*	Permanent
F2	M	>150	1.7	Goitre	Severe	Homo	*SLC5A5/SLC5A5*	Permanent
F13	F	12	9.1	Eutopic	Moderate	Het	*TG*	Permanent
F16	M	>100	4.4	Eutopic	Severe	Het	*TG*	Permanent
F18	M	12	7	Eutopic	Moderate	Het	*TG*	Permanent
F24	M	150	5.6	Goitre	Moderate	Het	*TG*	Permanent
F29	M	34	14.3	Eutopic	Mild	Het	*TG*	Permanent
F31	M	26	14.2	Eutopic	Mild	Het	*TG*	Permanent
F33	F	95	11.2	Eutopic	Mild	Het	*TG*	Permanent
F10	M	128	7	Goitre	Moderate	Oligo	*TG/DUOX2*	Permanent
F20	F	300	4.4	Goitre	Severe	Oligo	*TG/DUOX2*	Permanent
F7	M	13	15	Goitre	Mild	Oligo	*TG/DUOX2*	Permanent
F9	M	62	10	Goitre	Mild	Oligo	*TG/DUOX2*	Permanent
F1	F	>500	4.3	Goitre	Severe	Homo	*TG/TG*	Permanent
F28	M	>180	3.7	Goitre	Severe	Homo	*TG/TG*	Permanent
F3	M	N/A	8.6	Goitre	Moderate	HetComp	*TG/TG*	Permanent
F4	M	173	5.1	Goitre	Moderate	HetComp	*TG/TG*	Permanent
F23	M	>100	1.6	Goitre	Severe	Oligo	*TG/TPO*	Permanent
F8	F	700	1.2	Goitre	Severe	Oligo	*TG/TPO*	Permanent
F22	F	N/A	11.9	Eutopic	Mild	Het	*TPO*	Permanent
F25	F	29	13.6	Eutopic	Mild	Het	*TPO*	Permanent
F5	M	13	18.2	Eutopic	TSH resistance	Het	*TPO*	Permanent
F19	F	>100	2	Goitre	Severe	HetComp	*TPO/TPO*	Permanent
F26	F	>150	2.9	Eutopic	Severe	Homo	*TPO/TPO*	Permanent
F27	F	N/A	8.7	Goitre	Moderate	Homo	*TPO/TPO*	Permanent
F35	M	24.2	12.6	Goitre	Mild	Homo	*TPO/TPO*	Permanent
F14	F	44	20.6	Hypo	TSH resistance	Het	*TSHR*	Permanent
F15	M	447	2.2	Eutopic	Severe	Het	*TSHR*	Permanent
F34	F	61	8.6	Goitre	Moderate	Het	*TSHR*	Permanent
F6	M	50	16.6	Hypo	TSH resistance	Het	*TSHR*	Permanent

Severity of disease is defined on FT4 levels at the age of 10 days according to ESPE consensus criteria in mild, moderate or severe (respectively in clear, gray, or black); severe, < 5 pmol/L; moderate, 5–10 pmol/L; mild, 10–15 pmol/L. Serum TSH at confirmation, IU/ml; FT4, pmol/L.

Hom, homozygous; Het, heterozygous; HetComp, compound heterozygous; Oligo, oligogenism; TG, thyroglobulin; TPO, thyroperoxidase; TSHR, TSH receptor; SLC5A5, sodium/iodide symporter (NIS); DUOX2, dual oxidase 2; N/A, not applicable.

## Discussion

Early TDH diagnosis and treatment are essential for normal brain development and physical growth, but genetic and carrier identification encourage familial counselling and can allow for delaying the decision to treat for 3 years. Over the past two decades, the increased incidence of TDH associated with the high heterogeneity of its biological spectrum has been investigated. Relative to the technical limitations of direct sequencing for diagnosis, recent studies have demonstrated the efficiency of next-generation sequencing technology for CH screening (4, 6, 8, 19, 20). Our study exploring 65 patients identified by the French neonatal screening program in one reference centre showed that, for predominantly non-consanguineous families of different ethnic origins, familial TDH was frequent in France (56.9%). Irrespective of biological CH severity, we also observed that goiter was mainly associated with familial TDH (56.7%) compared to infants negative for ACMG variant classes 3 to 5, as a relevant clinical sign to ask for a genetic investigation. As previously described, we identified that homozygous variants in *TG*, *TPO,* and *SLC5A5* and oligogenicity were associated with goiter and severe CH whereas monoallelic variants in *TSHR*, *TG*, *DUOX2,* and *TPO* were related to milder phenotypes (21, 22). Inactivating heterozygous TSHR variants were present in 10.8% (4/37 cases) of our TDH cases with a heterogeneous clinical presentation: CH severity was inconstant, especially for the hot spot variant Arg450His leading to both subclinical (F6) and severe CH (F15). These results were in accordance with previous recommendations for systematic molecular analysis of the *TSHR* gene for CH with TSH resistance presentation (23). Thus, genetic exploration of TDH should be now considered as necessary as that for thyroid dysgenesis after neonatal screening.

In our study, monoallelic *DUOX2* variants were as frequent as monoallelic *TPO* variants, but were also more frequently involved through an oligogenic mechanism. *DUOX2* variants are associated with high phenotypic variability from transient to permanent CH and sometimes there is a difference in CH severity for the same variant (24). We observed the same variability from severe to mild or transient CH for infants in our study without a convincing hypothyroidism being found in the carrier parent. The presence of two DUOX isoforms could constitute an efficient redundant mechanism to maintain sufficient H_2_O_2_ supply for iodide organification. Thus, transient CH could be related to the difference in thyroid hormone requirements from the neonatal period to adulthood with sufficient H_2_O_2_ supply by DUOX1 only after the infantile period (5, 25, 26).

The contribution of oligogenic variants was frequent (21.6%) in our study, with diverse CH severity. Our results are consistent with the recent study of de Filippis and colleagues presenting frequent oligogenic inheritance (26.2%) in CH patients with either gland-in-situ or dysgenesis (6). In such cases, heterozygous variants in several genes can lead to cross-loss of enzyme activity (27, 28). Thus, monoallelic variants in the *TG* gene suggest that the oligogenic mechanism could be related to previous questionable CH cases with apparently unique heterozygous variants in the *TG* gene on direct sequencing (29). By exploring oligogenic inheritance, we showed that this mechanism was responsible for TDH with a significant association with severe CH and goiter. This oligogenic model could also explain differences in the penetrance and expressivity of CH in some families.

Our NGS design was able to detect CNV, intronic and proximal regulatory variants in the chromosomal region of the genes explored. None of these anomalies was detected in our families. This modern genetic testing has demonstrated that complex mechanisms can be frequently involved in the phenotypic heterogeneity of CH, even if the presence of additional variants on non-explored chromosomes or epigenetic regulation could not be excluded (30). We recommend increasing the diagnostic efficiency of CH by screening a large panel of genes involved in thyroid genesis, hormone synthesis and action from the TDH diagnosis. In accordance with recent recommendations from the ENDO-European Reference Network (30), we confirm that targeted-NGS or WES analyses for documented families should be used to improve CH diagnosis and treatment. Thus, except for the *DUOX2* heterozygous variant, infants with TDH and positive for deleterious variants did not need a diagnostic re-evaluation between two- and three-years age.

## Data Availability Statement

The data for this study have been deposited in the European Nucleotide Archive (ENA) at EMBL-EBI under accession number PRJEB42793.

## Ethics Statement

The studies involving human participants were reviewed and approved by DC-2015-2450 Biological Resource Center of Toulouse Hospital. Written informed consent to participate in this study was provided by the participants’ legal guardian/next of kin.

## Author Contributions

All authors provided contributions to study conception and design, acquisition and interpretation of data, and drafting the article. Most contributions in design of the study: IO-P and FS. Data collection: IO-P, TE, MB, SG and AC. Analysis of data: FS and VJ. Manuscript writing: IO-P and FS. All authors contributed to the article and approved the submitted version.

## Funding

This work was supported by the European Intereg Poctefa Piprepred Project (2014-2020).

## Conflict of Interest

The authors declare that the research was conducted in the absence of any commercial or financial relationships that could be construed as a potential conflict of interest.
