# Supplementary material for: Next-Generation Sequencing Analysis Reveals Frequent Familial Origin and Oligogenism in Congenital Hypothyroidism With Dyshormonogenesis
Source: Front Endocrinol (Lausanne). 2021 Jun 24;12:657913. doi: 10.3389/fendo.2021.657913 (PMC8264654; doi:10.3389/fendo.2021.657913)
Supplement: Supplementary file 1 [file DataSheet_1.pdf]

Gene	Exons	NCBI reference	coverage (%)
**TG**	48	NM_003226.4	100
**TPO**	18	NM_000547.5	100
**DUOX1**	35	NM_017434.4	100
**DUOX2**	32	NM_54799.4	98.5
**DUOXA2**	6	NM_997464.2	99.2
**SLC5A5**	15	NM_000444.1	99.8
**SLC26A4**	21	NM_000441.1	99.1
**SLC16A2**	6	NM_006517.3	100
**IYD**	8	NM_203395.2	100
**TSHR**	10	NM_000369.2	100
**GNAS1**	13	NM_000516.5	100
**NKX2-1**	3	NM_003317.3	100
**NKX2-5**	2	NM_004387.3	100
**THRA**	10	NM_003250.5	100
**THRB**	10	NM_00461.4	100
**SECISBP2**	17	NM_024077.4	100
**DUOXA1**	10	NM_144565.3	100
**PAX8**	12	NM_003466.3	100
**FOXE1**	1	NM_004473.3	99.1

Supplemental Table 1 : List and coverage of the 19 genes included in the NGS panel used for the genetic CH screening
