# Supplementary material for: Next-Generation Sequencing Analysis Reveals Frequent Familial Origin and Oligogenism in Congenital Hypothyroidism With Dyshormonogenesis
Source: Front Endocrinol (Lausanne). 2021 Jun 24;12:657913. doi: 10.3389/fendo.2021.657913 (PMC8264654; doi:10.3389/fendo.2021.657913)
Supplement: Supplementary file 2 [file DataSheet_2.pdf]

ID	Sex	Serum TSH (confirmation)	Serum FT4	Thyroid Evaluation	CH Severity	Permanent/transient
F38	F	25	11.8	Eutopic	Mild	Transient
F39	F	20	12.9	Eutopic	Mild	Transient
F40	M	20	6.2	Eutopic	Moderate	Transient
F41	M	43	5.3	Eutopic	Moderate	Transient
F42	M	56	10.7	Goitre	Mild	Transient
F43	M	65	15	Goitre	Mild	Permanent
F44	M	45	7	Goitre	Moderate	Permanent
F45	M	25	13.4	Eutopic	Mild	Transient
F46	M	62	11.5	Goitre	Mild	Transient
F47	F	22	7.2	Eutopic	Moderate	Permanent
F48	F	66	6.2	Eutopic	Moderate	Permanent
F49	F	37	14.6	Goitre	Mild	Permanent
F50	F	78	13	Goitre	Mild	Transient
F51	M	30	8.6	Eutopic	Moderate	Permanent
F52	M	22	5.1	Eutopic	Moderate	Permanent
F53	F	28	8.7	Eutopic	Moderate	Transient
F54	M	45	8.6	Eutopic	Moderate	Transient
F55	M	29	7	Eutopic	Moderate	Transient
F56	M	12	5.4	Eutopic	Moderate	Transient
F57	M	29	5.9	Eutopic	Moderate	Transient
F58	F	43	6.2	Eutopic	Moderate	Transient
F59	F	67	7.2	Eutopic	Moderate	Transient
F60	F	55	8.6	Eutopic	Moderate	Transient
F61	F	23	7	Eutopic	Moderate	Permanent
F62	F	20	9.1	Eutopic	Moderate	Permanent
F63	F	12	7.4	Eutopic	Moderate	Permanent
F64	M	18	8.1	Eutopic	Moderate	Permanent
F65	F	27	8.4	Eutopic	Moderate	Transient

Supplemental Table 2: Clinical and biological data for the 28 children diagnosed for TDH without identified variant.

Severity of disease is defined on FT4 levels at the age of 10 days according to ESPE consensus criteria in mild, moderate or severe (respectively in clear, grey or black); severe < 5 pmol/L; moderate: 5-10 pmol/L; mild : 10-15 pmol/L. Serum TSH at confirmation : IU/ml; FT4 : pmol/L.
